# Implanted Miniaturized Antenna for Brain Computer Interface Applications: Analysis and Design

**DOI:** 10.1371/journal.pone.0103945

**Published:** 2014-07-31

**Authors:** Yujuan Zhao, Robert L. Rennaker, Chris Hutchens, Tamer S. Ibrahim

**Affiliations:** 1 Department of Bioengineering, University of Pittsburgh, Pittsburgh, Pennsylvania, United States of America; 2 Behavioral and Brain Sciences, Erik Jonsson School of Engineering, University of Texas Dallas, Richardson, Texas, United States of America; 3 School of Electrical and Computer Engineering, Oklahoma State University, Stillwater, Oklahoma, United States of America; 4 Department of Radiology, University of Pittsburgh, Pittsburgh, Pennsylvania, United States of America; Institute for Frontier Medical Sciences, Kyoto University, Japan

## Abstract

Implantable Brain Computer Interfaces (BCIs) are designed to provide real-time control signals for prosthetic devices, study brain function, and/or restore sensory information lost as a result of injury or disease. Using Radio Frequency (RF) to wirelessly power a BCI could widely extend the number of applications and increase chronic in-vivo viability. However, due to the limited size and the electromagnetic loss of human brain tissues, implanted miniaturized antennas suffer low radiation efficiency. This work presents simulations, analysis and designs of implanted antennas for a wireless implantable RF-powered brain computer interface application. The results show that thin (on the order of 100 micrometers thickness) biocompatible insulating layers can significantly impact the antenna performance. The proper selection of the dielectric properties of the biocompatible insulating layers and the implantation position inside human brain tissues can facilitate efficient RF power reception by the implanted antenna. While the results show that the effects of the human head shape on implanted antenna performance is somewhat negligible, the constitutive properties of the brain tissues surrounding the implanted antenna can significantly impact the electrical characteristics (input impedance, and operational frequency) of the implanted antenna. Three miniaturized antenna designs are simulated and demonstrate that maximum RF power of up to 1.8 milli-Watts can be received at 2 GHz when the antenna implanted around the dura, without violating the Specific Absorption Rate (SAR) limits.

## Introduction

Brain Computer Interfaces (BCIs) are devices designed to establish a communication link between the human brain and neuroprosthetic devices to assist individuals with neurological conditions. However, because of the limitation of the power supply, most BCIs require a direct power connection with the external devices. The BCIs could be only implanted inside the subjects’ brain for a very limited time, which limit BCIs’ functionality and therefore limit the applications of clinical practice.

Battery can be used as BCI power supply units [1]–[3]. However, batteries present significant challenges due to the size, mass, toxic composition, and finite lifetime. There are several research groups using the inductive coupling method to transfer the power wirelessly [4]–[6]. The coupling coils have been typically designed to operate at 10 MHz or below (quasi-static conditions). The drawback of the inductive coupling is that its transmission mainly depends on the changing of magnetic field flux, which requires a relatively large (diameter of several centimeters) implanted coil precisely aligned with an external coil. The distance between two coupling coils is limited to approximately one centimeter in order to maintain the effective coupling results [7].

There are some groups studying implanted antennas to transmit data wirelessly into the human body. Most of these implanted antennas have been designed to operate at the medical implant communication service (MICS) band of 402–405 MHz. The implantable small profile patch antennas’ characteristics and their radiation were evaluated [8], [9]. The transmission and reflection of microstrip antennas affected by different superstrates and substrates were studied [10], through numerical analysis and measurements. The effects of different inner insulating layers and external insulating layers and power loss were discussed [11] analytically, using a spherical model. Besides the radiation efficiency impacts of insulating layers were presented [12]. For GHz and above operating frequencies, the impact of the coating on antenna performance was studied by an implanted antenna radiation measurement setup [13]. A pair of microstrip antennas working at microwave frequencies (1.45 GHz and 2.45 GHz) established a data telemetry link for a dual-unit retinal prosthesis [14].

Recent research reveals that the electromagnetic field penetration depth inside the tissue can be asymptotically independent of frequency at high frequencies, and the optimal frequency for the millimeter sized implanted antennas is in the gigahertz range. [15] An implanted antenna operating in the gigahertz range could be designed into a very small profile and also solve the difficulties in designing efficient high data rate [16]. Therefore, an implanted antenna (operating in the gigahertz range) provides a promising approach to accomplish a long term implantation of BCI in users as well as transmits power effectively.

Most of the abovementioned works are assuming that the implanted antennas are connected with 50 Ohm transmission lines. It is noted however, that the ratio between received RF power and tissue absorption depends on the input impedance of the receive antenna [15]. To realize the conjugate matching (i.e. optimal performance), the antenna loads including connected wires and implanted chips could be designed to other values rather than being restricted to 50 Ohms. For example the optimal choice was a 5.6 Ohms load in Poon’s study [15]. In our work, we simulate and characterize the input impedance of the implanted BCI RF power receiving antenna operating at an RF above 1 GHz. The input impedance and efficiency of wireless implanted antenna is evaluated for different 1) thickness of insulating layers 2) dielectric properties of insulating layers 3) location of implants, and 4) tissue compositions. Lastly, three miniaturized implanted antenna designs are compared and the maximum received power under the SAR regulations are calculated based on the FDTD simulation results.

## Materials and Methods

### FDTD simulation and the transmission line feed model

The input impedance of an antenna of the classic structure could be calculated analytically when the antenna is placed in the free space, buried in materials [17], or even when insulated antenna is embedded inside a homogeneous lossy material [18]. However, it is extremely challenging to analytically calculate the impedance of an insulated antenna with arbitrary structures embedded in the human brain, which integrates many different lossy tissue materials.

The FDTD method has great advantages for simulating interactions of electromagnetic waves with biological tissues [19]. In this work, a one dimensional transmission line feed model [2], [20] is implemented into our in-house three dimensional (3D) FDTD method package in order to study the input impedance of the implanted antenna. This simulation package has been widely utilized and verified in many papers [21]–[24]. The perfectly matched layers (PML) are used as the absorbing boundary conditions and the power radiated from the antenna in the FDTD model propagates similarly as it does in the lossless/lossy medium of infinite extent. The material of the antenna is simulated as the perfect electric conductor (PEC) to model very good conducting materials. To get the accurate computational results, the integration contour of the currents is shifted one cell from the antenna drive point to avoid the electric fringing field in the gap [2]. To analyze the ultra-thin (micrometers) insulating layers effects on the antennas performance, thin material sheets are modeled using a three dimensional sub-cell modeling formula in FDTD [25]. This efficient sub-cell modeling method removes the limitation that spatial information should be much larger than the cell grid and therefore greatly reduce the computer storage requirement and computational time.

At the feeding location, the antenna is excited by the virtual transmission line [26], which is injected with a differentiated Gaussian pulse with sufficient frequency content around the intended operational frequency. The differentiated Gaussian pulse is:
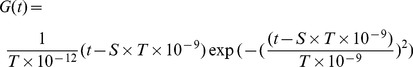
(1)


The parameter T affects the pulse-width and the time delay of the pulse. S is a temporal delay parameter. A set of suitable parameters for S (5.8) and T (0.1) have been chosen for a wideband spectrum of frequencies ranging from 1 GHz to 4 GHz according to the geometries of the antennas to be simulated.

### Antenna geometry and antenna performance parameters

The antenna reciprocity theorem [27] guarantees that a good transmitting antenna is also a good receiving antenna. The transmission/radiation efficiency is in part proportional to the radiation resistance [12], [27]. Generally for one specific antenna design, the radiation resistance of the antenna increases when the antenna size is larger [28]. In addition, the chip circuitry (attached to the implanted antenna) typically possesses high input impedance values (∼80–200 Ohms). Therefore, for efficient operation (minimal mismatch), it is highly favorable to have the input impedance of the implanted antenna in the same range (∼80–200 Ohms). The input impedance of a folded dipole antenna is approximately four times of the impedance of a dipole antenna when the length of the folded dipole equals to half wavelength [27], which is on the order of about 300 Ohm in the free space. As a result, a modified folded dipole antenna (rectangular antenna) was chosen for the following analysis.

Due to the inhomogeneous and lossy environment (human head), the relation between power reception and the implantation depth of the antenna does not strictly follow the Friis transmission formula as it is not a far field RF problem. Therefore the radiation pattern is not used to study the antennas’ performance in this work. Since the RF power is absorbed by the body and can result in tissue heating, the major concern about the wireless powering the BCI devices is mainly related to this safety issue. As a result, the main performance parameter of the BCI implanted antennas mainly depends on power reception in relation to tissue absorption i.e. SAR rise. Thus any geometry/feeding design of the antenna will aim at achieving maximum power reception for a given local SAR. Furthermore, from circuit theory, a maximum transfer of power from a given voltage source to a load occurs when the load impedance is the complex conjugate of the source impedance. Therefore, the input impedance of the implanted antenna is studied as the major power transmission indicator. The antennas can be used at any frequency where they exhibit enough power receptivity for a given local SAR. The input impedance and the received power of the implanted antenna are calculated through voltage and current information from the transmission line feed model [2], [20] used in this study.

### Human Head model

**Figure 1:**
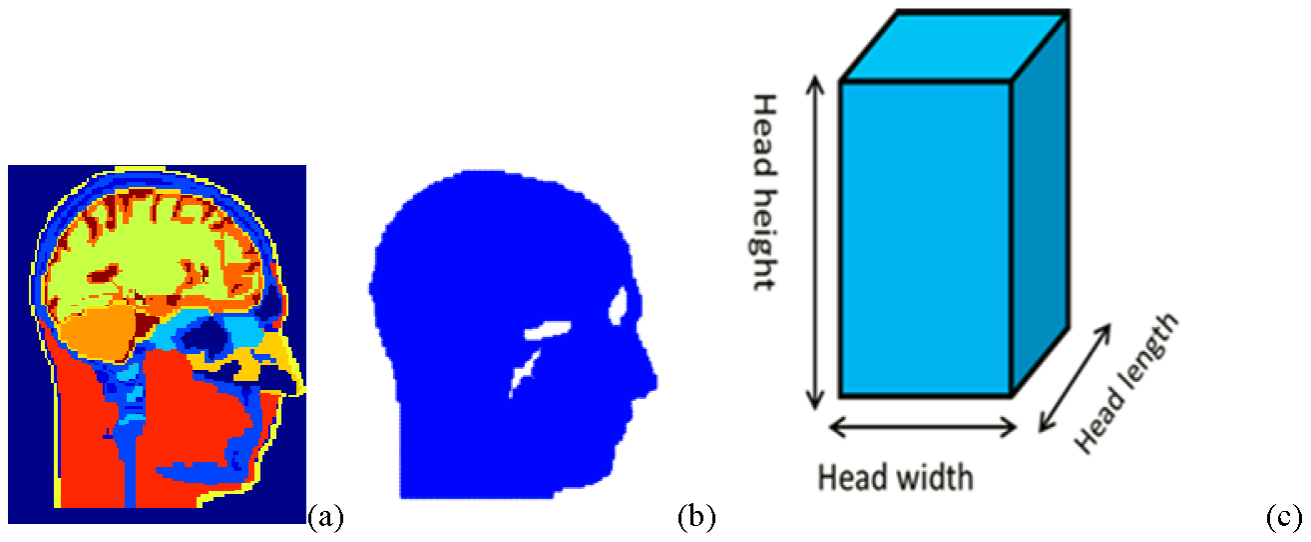
Phantoms and head models. a) Sagittal cross sections of the multi tissues head model at the middle slice; b) Sagittal cross section of the homogenous head shape phantom model at the middle slice; c) Homogeneous rectangular shape phantom model.

Antennas are implanted inside a 3D 19 materials head model which is developed from 1.5 tesla MRI images [29]. The tissue properties are defined [24] based on the study [30]. In order to compare the different effects of phantoms and the head model, two phantoms (different shapes) with the same single tissue material are also implemented, which are shown in Figure 1. The size of the head model/phantom is 182 mm×187 mm×230 mm. The implantable electrode arrays are normally implanted inside the cortex and the processing chip is between the dura and the grey matter [1]. Therefore, the dielectric properties of these two single-tissue head phantoms are calculated from the average of properties of the dura and the grey matter [30] (relative permittivity of 46 and conductivity of σ = 1.6 S/m).

## Results

### Effects of ultra-thin insulating layers on the input impedance of the implanted antennas

Biocompatible insulating materials are used to surround implanted antennas in order to prevent metallic oxidation and avoid the short circuit effect from the high conductive human head tissues. These biocompatible insulating layers could even the electromagnetic wave transition between the source and the head model and reduce the coupling with the lossy human tissues [11]. From the antenna miniaturization techniques aspect, the dielectric loading (biocompatible insulating material) has also been shown to be a very effective way of reducing the dimensions of the antenna [31]. Furthermore, the tissue model in the area immediately surrounding the implant affects the antenna performance considerably [9]. In this work, the impacts from the micrometer scale insulating layers are studied.

**Figure 2:**
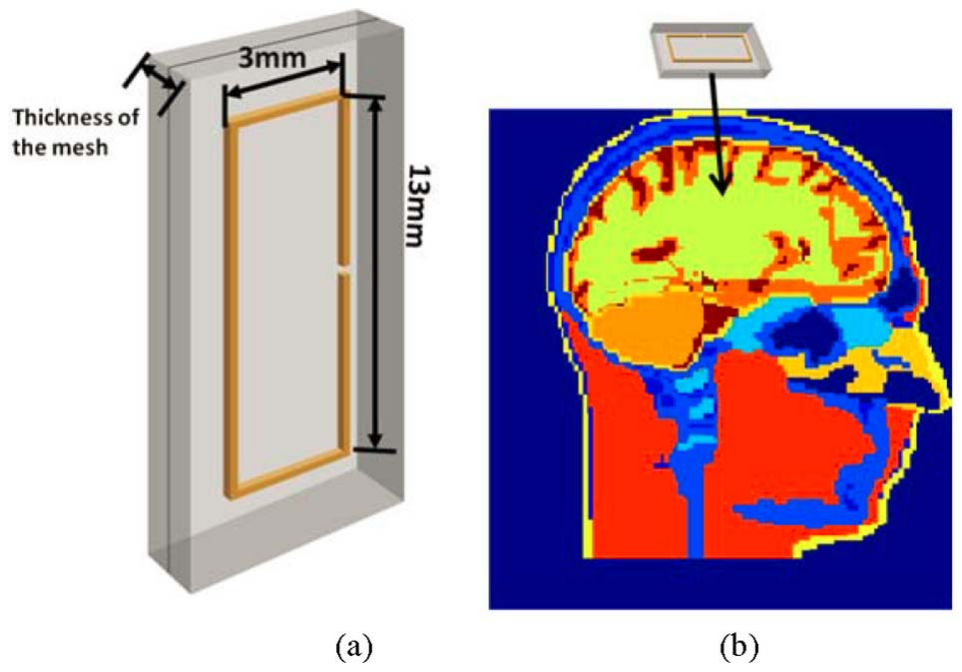
Simulated antenna geometry and its location. a) Geometry of the implanted rectangular antenna; b) Antenna position inside the head model (sagital view of the head model is shown), the color bar scale represents the relative permittivity values.

Description of the rectangular with a length of 13 mm and width of 3 mm (the thickness and width of the wire of this implanted antenna is negligible) surrounded by the insulating layer is shown in Figure 2a. In the Figure 2a, the dark rectangular line is the antenna wire and the grey part is the biocompatible insulating material mesh. The excitation is located at one of the longer parallel wires. The antenna surrounded by the insulating layer is numerically implanted into the center of the brain of the 3D anatomically detailed human head model (Figure 2b).

The simulation spatial resolution is set to 1 mm in this study. The thicknesses of the insulating layers are changing from 25 um to 330 um (thin material sheets are modeled using the three dimensional sub-cell modeling formula in FDTD [25]). Since the biocompatible materials are usually polymers and ceramics which are low conductive materials, the relative permittivity of the insulating layers is simulated as 2.1 (polycarbonate) in this simulation and the conductivity is approximately zero [32], [33].

**Figure 3:**
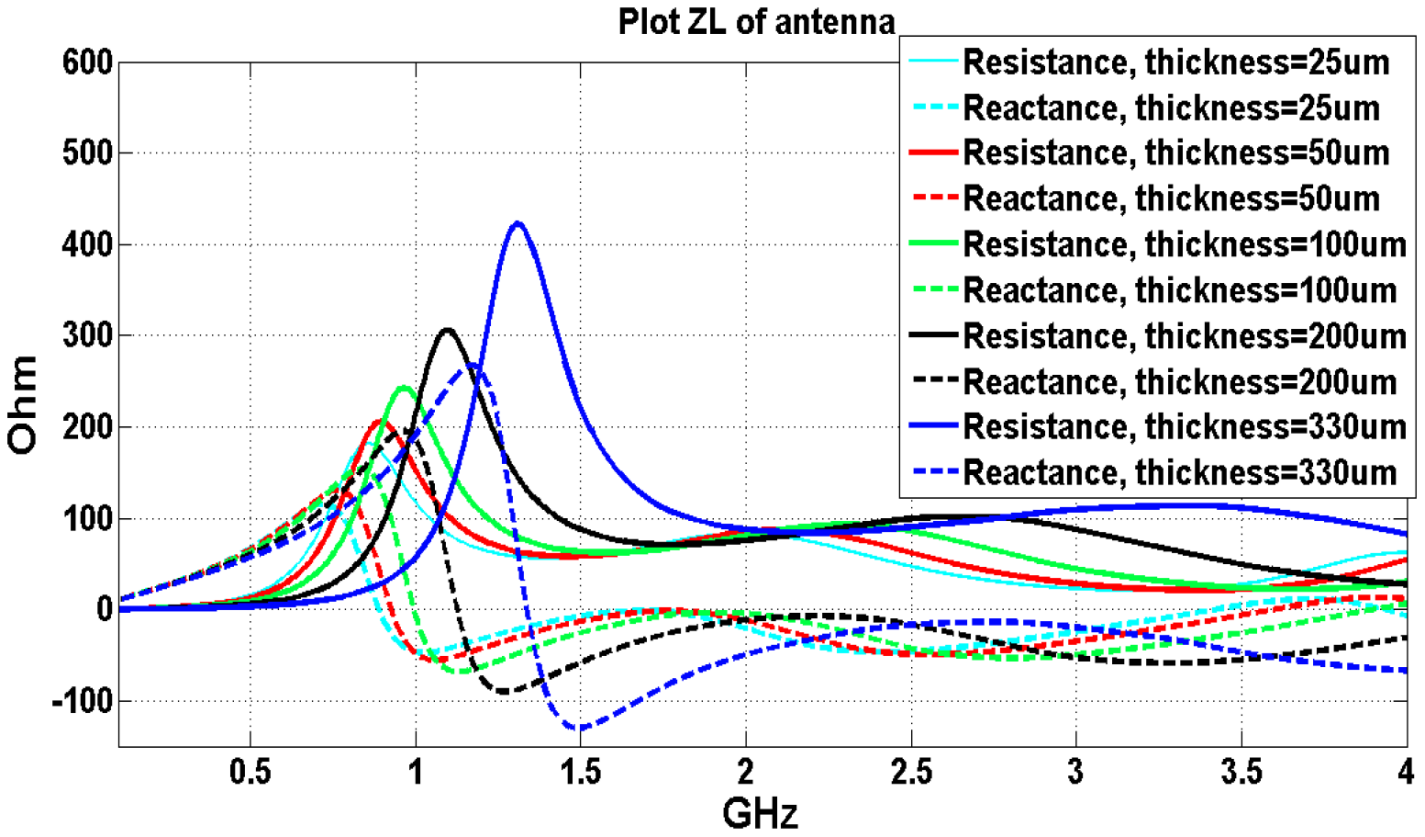
Effects of thin insulating layers on the input impedance of the implanted antenna inside the head model.

The results in Figure 3 demonstrate that the thickness of insulating layers significantly impacts antenna’s resonance frequency and input impedance, which in turn will affect antenna’s radiation efficiency. The results could be explained: when an antenna is implanted inside the human head model, the dielectric constant of insulating layers (2.1 in this case), is much smaller than that of the head tissues. The velocity of the electromagnetic wave is higher in the small dielectric constant material thus yielding longer operating wavelength. Therefore the resonant frequency of the same length antenna will shift to higher frequency when compared to non-insulating cases. This effect increases when the insulating layer becoming thicker (from 25 um to 330 um). The real part of the input impedance also increases because of the decreased average dielectric constant of the whole surrounding volume of the implanted antenna, including the insulating material and the brain tissues. In other words, the lossy human tissue material is moved away from the near field of the implanted antenna with a micrometer insulating layer which will lead to higher radiation efficiency. For example, the 330 um insulating layer antenna real part of the input impedance (which is 420 Ohm ) more than doubles that obtained with the 25 um insulating layer antenna (which is 180 Ohm) as shown in Figure 3.

From the simulation results plot of the frequency and input impedance in Figure 3, the input impedance values don’t change dramatically for insulating layers with different thickness if the operating frequency is larger than the resonant frequency (1.7 GHz−4 GHz). Therefore, for this implanted antenna, if operational frequency is chosen at this frequency band, the mismatch from the thicknesses changing will be minimal.

### Effects of the insulating layer dielectric properties on the input impedance of the antennas

**Figure 4:**
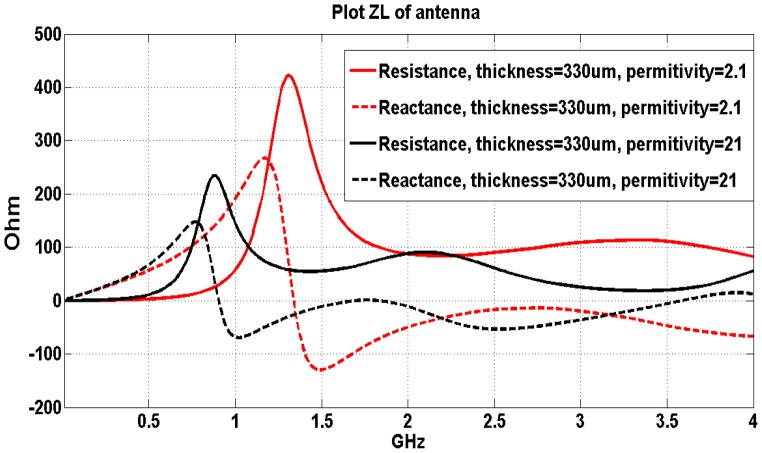
Simulation results of antennas surrounded with insulating layers with the same thickness but the different dielectric properties.

The same geometry of the rectangular implanted antenna shown in Figure 2a is simulated with two different biocompatible insulating layers (the simulated insulating layers have the same thickness of 0.33 mm in the two simulations) inside the human head model. The simulation results are shown in Figure 4.

The simulation results in this section show not only that the thickness of the insulating material affects antenna performance, but also the dielectric property of the insulating materials influence the performance of the implanted antenna inside the human brain. The results reveal that the antenna resonant frequency shifts to a lower frequency when the antenna is embedded inside a high dielectric constant insulating layer. Figure 4 also shows that the first resonant frequency is around 1.4 GHz if the relative permittivity is 2.1. If the antenna is embedded in the material with relative permittivity of 21, the center resonant frequency will be around t 0.9 GHz. Higher averaged dielectric constant of the media surrounding the antenna reduces the wavelength of the electromagnetic waves inside the media. As the length of the antenna depends on the wavelength of the antenna’s operational frequency; High dielectric constant insulating layer consequently facilitates the reduction of antennas geometric dimensions. However, high dielectric constant insulating layer may reduce the real part of the input impedance of the antenna which in turn may hamper the radiation efficiency. Therefore, a balance design of high radiation efficiency and smaller dimensions is crucial to achieve optimal performance.

### Effects of the head tissues properties on input impedance of implanted antennas

**Figure 5:**
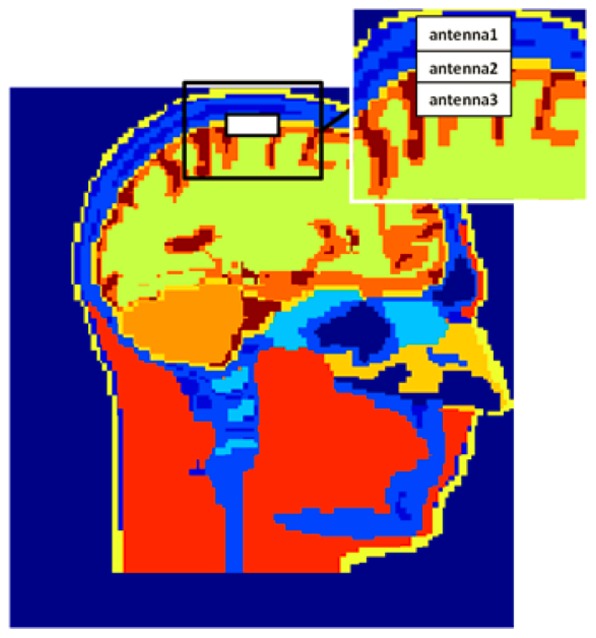
Implanted antenna at three different locations inside the human head model.

The performance of the implanted antenna is influenced by all surrounding materials which includes the biocompatible insulating layers and the lossy human head tissues. In this section, the same rectangular antenna is simulated at three different locations inside the human brain model. For the clinical usage, the BCI devices are normally implanted between the dura and the grey matter [1]. Hence, the three different locations are all proposed around the dura which is responsible for keeping in the cerebrospinal fluid. In Figure 5, the dura is represented by the light orange color around the brain cortex. Above the dura is the cortical bone and below the dura are the combination tissues of the dura and grey matter in the head model. Their constitutive properties and the simulated antenna positions in this head model are listed in Table 1. The same insulating layer (thickness of 1 mm and relative permittivity of 2.1) is used for three different simulation cases.

**Table 1:** Dielectric property of three adjacent major tissues at three different locations inside the human head (Fig. 6) at 2.4 GHz.

Tissue	Conductivity(S/m)	Relative permittivity
Bone Cortical (1.6 cm from the surface)	0.385	11.410
Dura (1.9 cm from the surface)	1.639	42.099
Brain Grey Matter (2.24 cm from the surface)	1.773	48.994

**Figure 6:**
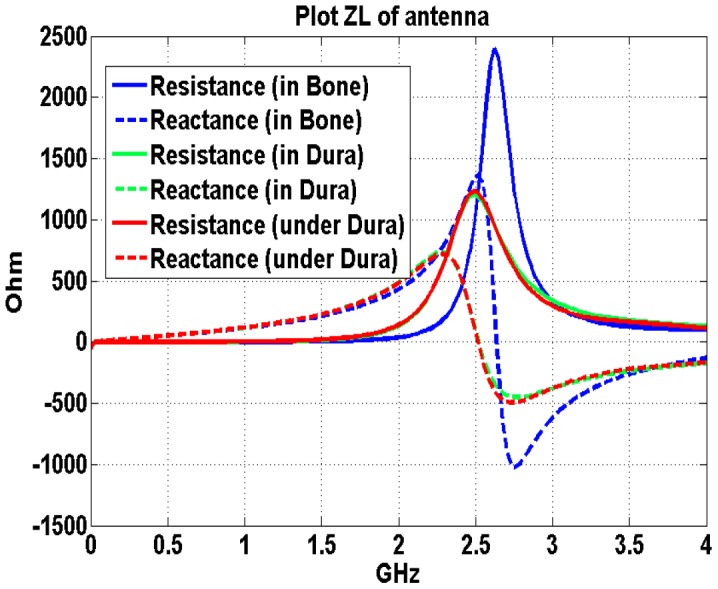
Input Impedance of the implanted rectangular antenna at three different locations inside the human head (**Figure 5**).

Table 1 shows that at 2.4 GHz the conductivity and relative permittivity of grey matter (1.773 S/m and 48.994 respectively) are similar with the dura’s dielectric property (1.639 S/m and 42.099 respectively) and different for that of the bone(0.385 S/m and 11.4) [30]. These similarities and differences hold true for all other frequencies of interest. Figure 6 displays input impedance of the implanted antenna at the three different implanted positions inside the human brain shown in Figure 5.

Since these three implantation positions are adjacent to each other, we assume that any performance differences of the antenna are not caused by the implantation depth. The results show that the implanted antenna performs differently in bone and in the dura while the same antenna performs relative similar when the antenna is implanted in the dura and directly under the dura. In addition, the brain tissues are separated from the implanted antenna by the biocompatible insulating layers. The frequency shifts and the impedance varieties caused by the tissues properties changes are not as significant as the biocompatible insulating layers’ impacts.

The input impedance of the antenna implanted above the dura, where cortical bone is present, is larger than the other two cases. Therefore, the antenna implanted in low conductivity tissues (e.g. cortical bone) may facilitate the antenna radiation efficiency. In addition, the antenna frequency could be altered with time caused by the saline absorption [13] resulting in instability in the antenna performance. The brain tissues with properties are stable over time and less saline content (i.e. the cortical bones) may be preferable for antenna implantations from the considerations of antenna transmission efficiency as well as the RF circuit stabilization. This of course will impact the design and dimensions of the micro wires and applicability of the BCI.

### Effects of the human head phantom shape and dielectric properties on the implanted antennas

A head shape phantom with single liquid mixture was experimentally used by other groups to test the human head effects on the implanted antenna. For example, in [34] the return loss and transmission parameters were measured using a head shape phantom by Schmidt & Partner Engineering for the dosimetric assessment system. To answer whether a multi tissues head phantom is necessary for measuring the implanted antenna performance accurately, and whether a head shape phantom with one homogeneous material could be used to test implanted antenna performance (frequency bandwidth and input impedance), the antenna performance is studied inside three different 3D phantom models. We utilized a multi-tissue head model, a homogenous head model, and a rectangular phantom model, all of which have the same head height, length, and width (see Figure 1.) As mentioned, the relative permittivity is ε = 46 and conductivity is σ = 1.6 S/m for the rectangular phantom model and the homogenous head model.

The 3 mm by 12 mm rectangular antenna with 1 mm insulating layer is implanted 19 mm under the top of the multi tissues head model (Figure 1a) (the spatial resolution of the simulation is 1 mm), which is just under the dura of this head model. It is centered at the coronal and axial directions. The same insulated rectangular antenna is implanted at the exactly same physical positions inside the homogenous head shape phantom and the rectangular shape phantom model respectively.

**Figure 7:**
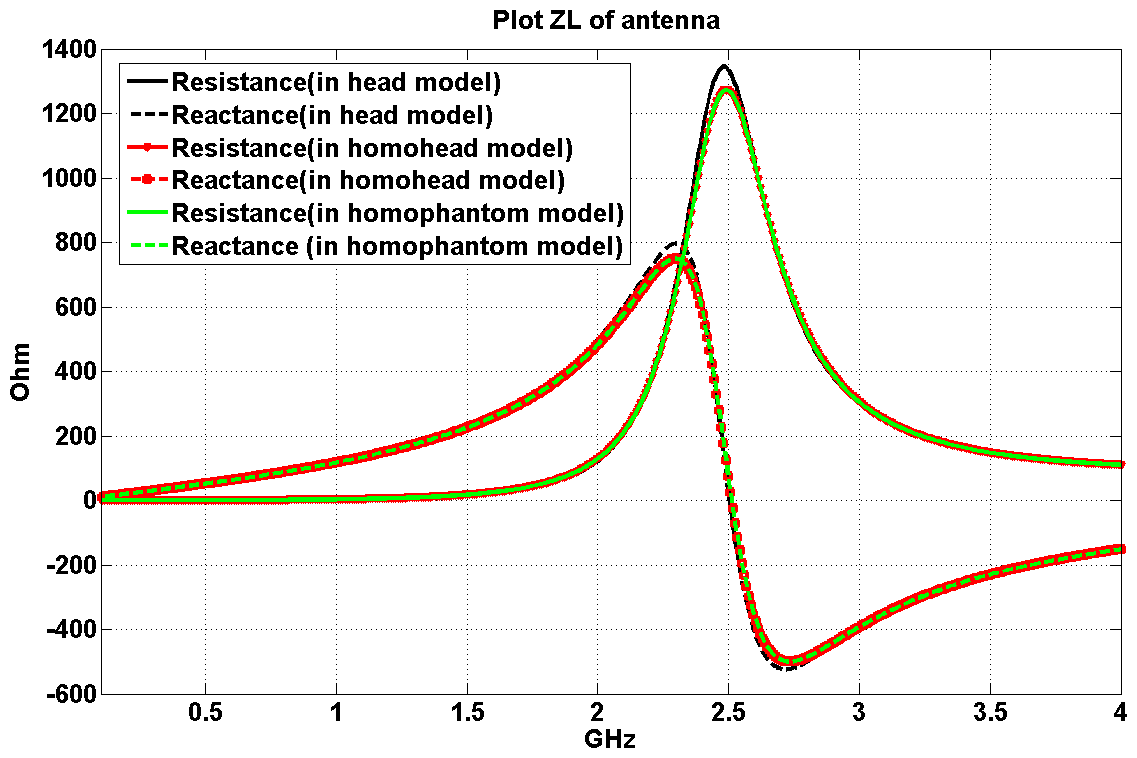
Input Impedance of the antenna when implanted 19-tissue head model, the head shape homogenous phantom model and the rectangular homogenous phantom model.

The simulation results are presented in Figure 7 and it demonstrates that the performances of the implanted antenna are highly similar inside the three head/phantom models, although the shapes of the head phantoms are different. Especially, the results are identical when the antenna is implanted inside the homo-head model and when it is inside the homo-phantom model. This verifies that the phantom model shape is not necessary to assess the implanted antenna’s performances (input impedance and resonance frequency) for this application. Rectangular homogenous phantom could be used instead of a more complex head shape phantom to assess the BCI implanted antenna’s specific characteristics (frequency band and input impedance).

While homogenous rectangular head-sized phantom could be used to study the implanted antenna’s bandwidth and input impedance, the head shape as well as the presence of different types of tissues is necessary to study heating/SAR/power transmission. This is because SAR as well as the power will change when RF waves go through different tissue, therefore the rectangular homogenous phantom may not be accurate to advise such information.

### Designs of the implanted antennas

Around 2.4 GHz, the minimum wavelength (15 mm) shows up in high water content material Cerebra Spinal Fluid (CSF) in human head tissues. Results of the one-cell-gap-feeding models show convergence to the true value if using fine grids [2], [35], so spatial resolution of 0.165 mm 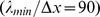 is implemented for the following miniaturized antenna designs. The time resolution of FDTD is calculated based on the stability conditions to satisfy the stability criterion.

Three implanted antenna designs are simulated and compared in this study. The same insulating material is used for these implanted antenna simulations (the thickness is 0.33 mm). The thickness of 0.33 mm is chosen because it is a feasible thickness to manufacture and assemble. The surrounding biocompatible material is peek [11] polymer (the relative permittivity is 3.2) which has excellent mechanical properties (stiffness, toughness and durability).

**Figure 8:**
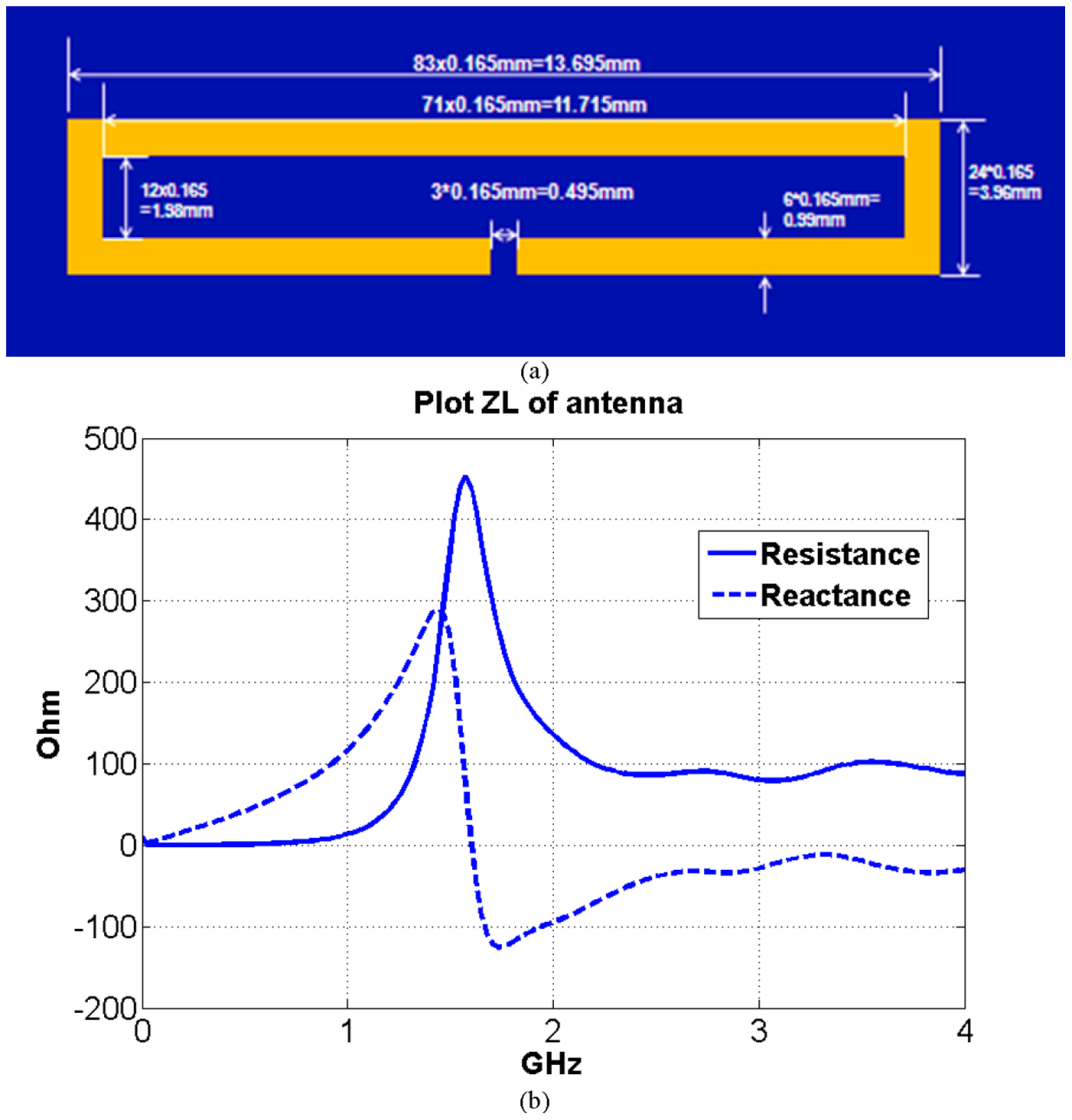
Implanted rectangular antenna, a) geometry and b) input impedance.

The first antenna design considered is a rectangular antenna. The detailed geometry is shown in Figure 8a. Its input impedance as a function of frequency was calculated using the FDTD model and is shown in Figure 8b. The first resonant frequency (when the imaginary part of the input impedance is zero) is around 1.6 GHz. In order to reduce the circuit mismatching effect, the frequency bandwidth could be chosen between 2 GHz and 4 GHz (because the impedance of the antenna is relative stable in this frequency band).

**Figure 9:**
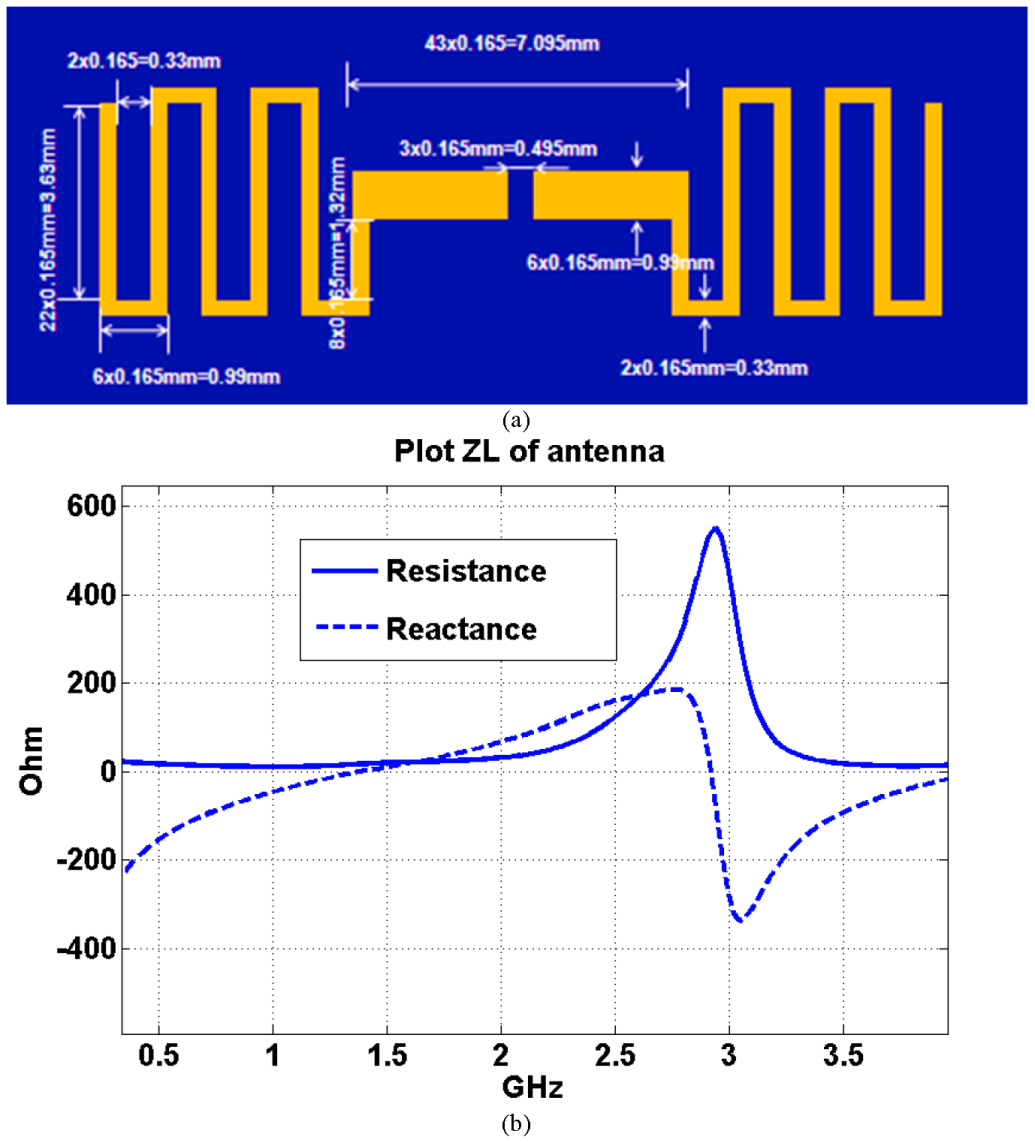
Implanted serpentine antenna, a) geometry and b) input impedance.

The second implanted antenna design considered is a serpentine antenna or a meander line antenna [36] which substantially has the greater length in a specific surface area. The geometry detail of the implanted serpentine antenna is shown in Figure 9a. The size of the implanted serpentine antenna (length of 13.695 mm and width of 3.96 mm) is almost the same as the length of the implanted rectangular antenna (length of 13.695 mm and width of 4.29 mm), but has a much longer physical wire length (55.935 mm for the serpentine antenna and 31.35 mm for the rectangular antenna). From the simulation results of the input impedance and frequency in Figure 9b, the first resonant frequency is around 1.38 GH, which is 220 MHz lower than the first resonant frequency of the implanted rectangular antenna. The frequency bandwidth could be chosen between 1 GHz and 2 GHz (the impedance of the antenna is relative stable in this frequency band). The real part of the input impedance of the serpentine antenna is almost one fifth of that associated with the rectangular antenna at their respective bandwidths (stable resistance slope as a function of frequency); 18 Ohm around 1.5 GHz for the serpentine antenna and 100 Ohm around 2.4 GHz for the rectangular antenna.

**Figure 10:**
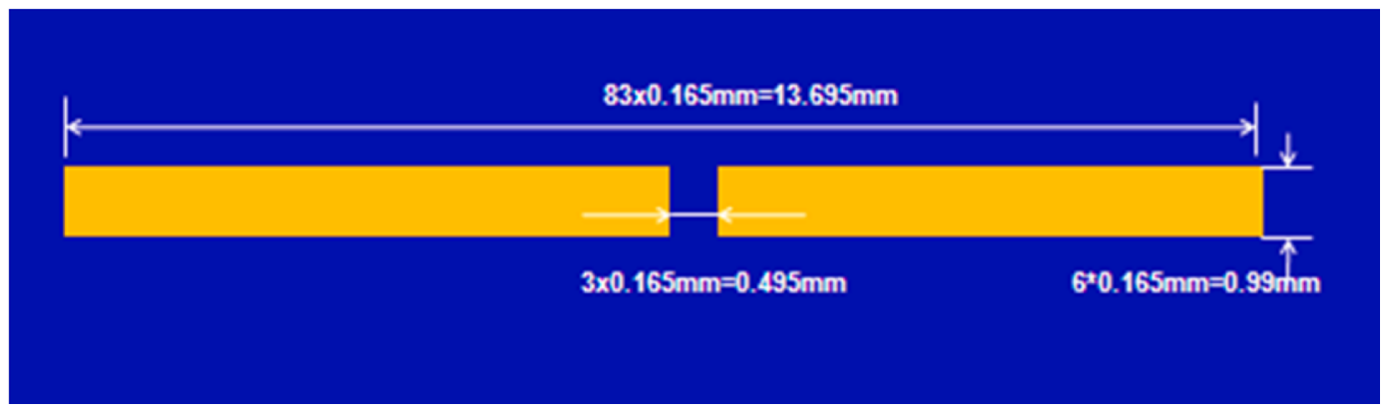
Geometry of the implanted dipole antenna.

The third implanted antenna design considered is a dipole antenna. The geometry detail of the implanted dipole antenna is shown in Figure 10. The first resonant frequency is around 5.2 GHz, which shows that the dipole antenna is electrically shorter than the other two antennas. Since the 5.2 GHz falls out of our accurate simulated range (1 GHz to 4 GHz), the impedance and frequency plot is not shown here. The real part of the input impedance around 2 GHz is around 14 Ohm.

### Maximum power reception without SAR violations

The SAR safety regulations regarding RF power deposition in the head varies for different applications. In this work, the power receptions of the implanted antennas are analyzed based on the IEEE RF safety Standard developed by the International Committee on Electromagnetic Safety (ICES) [37] (IEEE, 2005) and the International Commission on Non-ionizing Radiation Protection (ICNIRP) safety regulations [38] with respect to human exposure to radiofrequency electromagnetic fields up to 300 GHz. With respect to SAR limits, the frequency is from 100 kHz to 3 GHz in IEEE regulation and 100 kHz–10 GHz in ICNIRP regulation. According these two SAR regulations, the local SAR peak averaged over any 10 g of tissue in the head must be less than or equal to 2 W/kg.

In order to calculate the maximum power reception under the SAR limitations, a dipole antenna is chosen as the external transmitting antenna and the three different implanted antennas are simulated as the receiving antennas. Based on the analysis of these three designed antennas (especially the rectangular antenna and the serpentine antenna), the common preferred frequency band is around 2 GHz. Therefore, the length of the external antenna is defined as75 mm (with negligible thickness). Its resonant frequency is around 2 GHz (simulated and analyzed when the head model existing in the environment near the antenna). The distance between the transmit and receive antennas is about 30 mm; the inner antenna is just under the dura and the outside antenna is about 10 mm away from the surface of the head. Their excitation positions of transmit and receive antennas are vertically centered and placed at the same plane. The multi tissues head model is used to study the maximum received power from the implanted receiving antenna without violating the SAR limits.

Considering the implanted rectangular/serpentine/dipole antennas’ input impedance characteristics, the simulated load of implanted chip and circuits (virtual transmission line connected to the antenna ports) are modified to match with the real part of input impedance of the implanted receiving antenna at frequency 2.0 GHz. Considering there are also reactive parts, it is not a perfect match. Hence the calculated (in this work) maximum available power will represent a less optimized scenario: while the real part of impedance is identical for both the implanted receiving antenna and the chip circuitry/transmission lines, no matching circuit is utilized to compensate for the mismatch in the imaginary part. The calculated maximum power received by the three antenna designs at the SAR limit is shown in the Table 2. The results could be changed from the calculated results in this work (more power can be received potentially) once the source matched to the load perfectly.

**Table 2:** Maximum power reception under IEEE and ICNIRP SAR limit (2 Watts perKg per 10 gm) at 2 GHz when the implanted antenna is placed right under the dura.

Antenna	Maximum power reception (mW)
Rectangular antenna	1.3
Serpentine antenna	1.8
Dipole antenna	0.58

Table 2 shows the serpentine antenna allows for more power reception at the SAR limit than the rectangular antenna: the maximum received power is 1.8 mW at the SAR limit when the serpentine antenna is implanted around the dura. While the results show the superiority of the serpentine antenna in terms of power reception, the higher input impedance of the rectangular antenna allows for better interfacing with the typically expected high input impedance of the chip circuitry (less impedance mismatch).

Furthermore, the maximum power reception has also been investigated when the rectangular antenna implanted inside the cortical bone. The calculated result shows that the rectangular antenna implanted at the bone could receive about 2.5 times more RF power at the SAR limit than that obtained when the antenna is implanted at dura.

## Conclusion

Miniaturized antennas designs for the BCI application were simulated and analyzed in this work. The simulation results show that the micrometer thickness insulating layer can significantly impact implanted antenna performance. The proper selection of the dielectric properties of the biocompatible insulating layers and the implantation position inside head brain tissues would facilitate the RF power transmission/reception. The shape of the head model may be not a critical factor, but the dielectric properties of surrounding tissues can impact the implanted antennas’ input impedance and its operational frequency bandwidth.

Based on three miniaturized antenna designs’ simulation results, the maximum power of 1.8 mW could be received by an implanted serpentine antenna when it is implanted inside the dura at the IEEE and ICNIRP SAR limit. Assuming a 25% RF/DC conversion efficiency (due to the switching nature of the harvester circuits), the implantable BCI device can consume 450 uW or less based on the results in this work. Our current designs of simple implantable chip consume about 35 uW [39] which means the designed miniaturized antenna could provide sufficient power to this available chip design if placed in the dura.

## References

[1] Chestek CA, Gilja V, Nuyujukian P, Kier RJ, Solzbacher F, et al (2009) HermesC: low-power wireless neural recording system for freely moving primates. IEEE transactions on neural systems and rehabilitation engineering : a publication of the IEEE Engineering in Medicine and Biology Society 17: 330–338. 10.1109/TNSRE.2009.2023293 19497829

[2] Hertel TW, Smith GS (2003) On the convergence of common FDTD feed models for antennas. Ieee Transactions on Antennas and Propagation 51: 1771–1779.

[3] Farshchi S, Pesterev A, Nuyujukian P, Guenterberg E, Mody I, et al (2010) Embedded Neural Recording With TinyOS-Based Wireless-Enabled Processor Modules. Ieee Transactions on Neural Systems and Rehabilitation Engineering 18: 134–141. 20071270 10.1109/TNSRE.2009.2039606

[4] Song YK, Borton DA, Park S, Patterson WR, Bull CW, et al (2009) Active Microelectronic Neurosensor Arrays for Implantable Brain Communication Interfaces. Ieee Transactions on Neural Systems and Rehabilitation Engineering 17: 339–345. 19502132 10.1109/TNSRE.2009.2024310 PMC2921652

[5] Harrison RR, Kier RJ, Chestek CA, Gilja V, Nuyujukian P, et al (2009) Wireless Neural Recording With Single Low-Power Integrated Circuit. Ieee Transactions on Neural Systems and Rehabilitation Engineering 17: 322–329. 19497825 10.1109/TNSRE.2009.2023298 PMC2941647

[6] Irazoqui PP, Mody I, Judy JW (2005) Recording brain activity wirelessly. Ieee Engineering in Medicine and Biology Magazine 24: 48–54. 10.1109/memb.2005.1549730 16382805

[7] Kim S, Zoschke K, Klein M, Black D, Buschick K, et al (2007) Switchable polymer-based thin film coils as a power module for wireless neural interfaces. Sensors and Actuators a-Physical 136: 467–474. 10.1016/j.sna.2006.10.048 PMC2344127 18438447

[8] Kim J, Rahmat-Samii Y (2004) Implanted antennas inside a human body: Simulations, designs, and characterizations. Ieee Transactions on Microwave Theory and Techniques 52: 1934–1943.

[9] Kiourti A, Nikita KS (2013) Numerical assessment of the performance of a scalp-implantable antenna: effects of head anatomy and dielectric parameters. Bioelectromagnetics 34: 167–179. 22948753 10.1002/bem.21753

[10] Soontornpipit P, Furse CM, Chung YC (2004) Design of implantable microstrip antenna for communication with medical implants. Ieee Transactions on Microwave Theory and Techniques 52: 1944–1951.

[11] Merli F, Fuchs B, Mosig JR, Skrivervik AK (2011) The Effect of Insulating Layers on the Performance of Implanted Antennas. Ieee Transactions on Antennas and Propagation 59: 21–31.

[12] Hall PS, Hao Y (2006) Antennas and propagation for body-centric wireless communications. Boston: Artech House. 1 online resource (xiii, 291 s.) p.

[13] Warty R, Tofighi MR, Kawoos U, Rosen A (2008) Characterization of Implantable Antennas for Intracranial Pressure Monitoring: Reflection by and Transmission Through a Scalp Phantom. Ieee Transactions on Microwave Theory and Techniques 56: 2366–2376.

[14] Gosalia K, Lazzi G, Humayun M (2004) Investigation of a microwave data telemetry link for a retinal prosthesis. Ieee Transactions on Microwave Theory and Techniques 52: 1925–1933.

[15] Poon ASY, O’Driscoll S, Meng TH (2010) Optimal Frequency for Wireless Power Transmission Into Dispersive Tissue. Ieee Transactions on Antennas and Propagation 58: 1739–1750.

[16] Yakovlev A, Kim S, Poon A (2012) Implantable Biomedical Devices: Wireless Powering and Communication. Ieee Communications Magazine 50: 152–159.

[17] King RWP, Smith GS (1981) Antennas in Matter: Fundamentals, Theory, and Applications. Cambridge, MA: The MIT Press.

[18] Fenwick RC, Weeks WI (1963) Sumberged antenna characteristics. Ieee Transactions on Antennas and Propagation 11: 296–305.

[19] Ibrahim TS, Hue YK, Tang L (2009) Understanding and manipulating the RF fields at high field MRI. Nmr in Biomedicine 22: 927–936. 19621335 10.1002/nbm.1406 PMC4515035

[20] Taflove A, Hagness SC (2005) Computational electrodynamics : the finite-difference time-domain method. Boston: Artech House. xxii, 1006 p., [1008] f. de pl. en coul. p.

[21] Krishnamurthy N, Zhao T, Ibrahim TS (2013) Effects of receive-only inserts on specific absorption rate, B field, and Tx coil performance. J Magn Reson Imaging. 10.1002/jmri.24152 PMC4501854 23913474

[22] Tang L, Hue YK, Ibrahim TS (2011) Studies of RF Shimming Techniques with Minimization of RF Power Deposition and Their Associated Temperature Changes. Concepts Magn Reson Part B Magn Reson Eng 39B: 11–25. 21607117 10.1002/cmr.b.20185 PMC3098508

[23] Ibrahim TS, Tang L, Kangarlu A, Abraham R (2007) Electromagnetic and modeling analyses of an implanted device at 3 and 7 Tesla. Journal of Magnetic Resonance Imaging 26: 1362–1367. 17969135 10.1002/jmri.21148

[24] Zhao Y, Tang L, Rennaker R, Hutchens C, Ibrahim TS (2013) Studies in RF Power Communication, SAR, and Temperature Elevation in Wireless Implantable Neural Interfaces. PLoS One 8: e77759. 24223123 10.1371/journal.pone.0077759 PMC3819346

[25] Maloney JG, Smith GS (1992) The Efficient Modeling of Thin Material Sheets in the Finite-Difference Time-Domain (Fdtd) Method. Ieee Transactions on Antennas and Propagation 40: 323–330.

[26] Maloney JG, Shlager KL, Smith GS (1994) A Simple FDTD Model for Transient Excitation of Antennas by Transmission Lines. IEEE TRANSACTIONS ON ANTENNAS AND PROPAGATION 42: 289–292.

[27] Balanis CA (2005) Antenna theory : analysis and design. Hoboken, NJ: John Wiley. xvii, 1117 p. p.

[28] Endo T, Sunahara Y, Satoh S, Katagi T (2000) Resonant frequency and radiation efficiency of meander line antennas. Electronics and Communications in Japan Part Ii-Electronics 83: 52–58.

[29] Ibrahim TS, Lee R, Baertlein BA, Abduljalil AM, Zhu H, et al (2001) Effect of RF coil excitation on field inhomogeneity at ultra high fields: A field optimized TEM resonator. Magnetic Resonance Imaging 19: 1339–1347. 11804762 10.1016/s0730-725x(01)00404-0

[30] Andreuccetti D, Fossi R, Petrucci C (1997) An Internet resource for the calculation of the dielectric properties of body tissues in the frequency range 10 Hz–100 GHz. Website at http://niremf.ifac.cnr.it/tissprop/, IFAC-CNR, Florence (Italy). pp. Based on data published by C.Gabriel et al. in 1996.

[31] Skrivervik AK, Zurcher JF, Staub O, Mosig JR (2001) PCS antenna design: The challenge of miniaturization. Ieee Antennas and Propagation Magazine 43: 12–26.

[32] Alberti G, Casciola M, Massinelli L, Bauer B (2001) Polymeric proton conducting membranes for medium temperature fuel cells (110–160°C). Journal of Membrane Science 185: 73–81.

[33] Kobayashi T, Rikukawa M, Sanui K, Ogata N (1998) Proton-conducting polymers derived from poly(ether-etherketone) and poly(4-phenoxybenzoyl-1,4-phenylene). Solid State Ionics 106: 219–225.

[34] Chen ZN, Liu GC, See TSP (2009) Transmission of RF Signals Between MICS Loop Antennas in Free Space and Implanted in the Human Head. Ieee Transactions on Antennas and Propagation 57: 1850–1854.

[35] Zhao HP, Shen ZX (2009) Weighted Laguerre Polynomials-Finite Difference Method for Time-Domain Modeling of Thin Wire Antennas in a Loaded Cavity. Ieee Antennas and Wireless Propagation Letters 8: 1131–1134.

[36] Nakano H, Tagami H, Yoshizawa A, Yamauchi J (1984) Shortening Ratios of Modified Dipole Antennas. Ieee Transactions on Antennas and Propagation 32: 385–386.

[37] IEEE (2005) IEEE Standard for Safety Levels with Respect to Human Exposure to Radio Frequency Electromagnetic Fields, 3 kHz to 300 GHz. IEEE Standard for Safety Levels with Respect to Human Exposure to Radio Frequency Electromagnetic Fields, 3 kHz to 300 GHz.

[38] ICNIRP (1998) Guidelines for limiting exposure to time-varying electric, magnetic, and electromagnetic fields (up to 300 GHz). International Commission on Non-Ionizing Radiation Protection. Health Phys. 494–522. 9525427

[39] Hutchens C, Rennaker RL 2nd, Venkataraman S, Ahmed R, Liao R, et al (2011) Implantable radio frequency identification sensors: wireless power and communication. Conference proceedings : Annual International Conference of the IEEE Engineering in Medicine and Biology Society IEEE Engineering in Medicine and Biology Society Conference 2011: 2886–2892. 10.1109/IEMBS.2011.6090796 PMC3582674 22254944

